# Laparoscopic management of left paraduodenal hernia

**DOI:** 10.4103/0972-9941.72601

**Published:** 2010

**Authors:** B P S Parmar, R S Parmar

**Affiliations:** Department of Surgery, Parmar Nursing Home, Ropar, Punjab, India

**Keywords:** Paraduodenal hernia, laparsocopy, internal hernia

## Abstract

Internal herniation of small bowel accounts for about 1% of all the patients with intestinal obstruction. Fifty percent of the patients with paraduodenal hernia will have bowel obstruction. Left paraduodenal hernia resulting from abnormal rotation of the midgut during embryonic development is the most common form of congenital internal hernia. A case of a young male presenting with chronic abdominal pain due to left paraduodenal hernia is being reported. A correct preoperative diagnosis of left paraduodenal hernia was made on computerised tomography (CT), and the patient was managed by laparoscopic surgery. The role of imaging in preoperative diagnosis is being highlighted with a brief review of literature.

## INTRODUCTION

Amongst the congenital internal hernias, paraduodenal hernias are the most common and account for 25% to 53% of all cases.[1] Although internal hernias occur in only 1% of all patients with intestinal obstruction, 50% of patients with paraduodenal hernia have obstruction.[2] Advent of modern imaging techniques like computerized tomography has increased the probability of correct preoperative diagnosis of paraduodenal hernia, which was once difficult because of its non-specific presentation. We present a patient with a left paraduodenal hernia presenting with chronic abdominal pain managed successfully by laparoscopic surgery. A brief review of the literature is presented.

## CASE REPORT

A 38-year-old man presented with a 5-year history of intermittent left upper abdominal pain that was sometime precipitated by food and usually resolved spontaneously. There was no change in the bowel habit and no rectal bleeding. There was no past history of any other medical or surgical problems.

**Figure 1:**
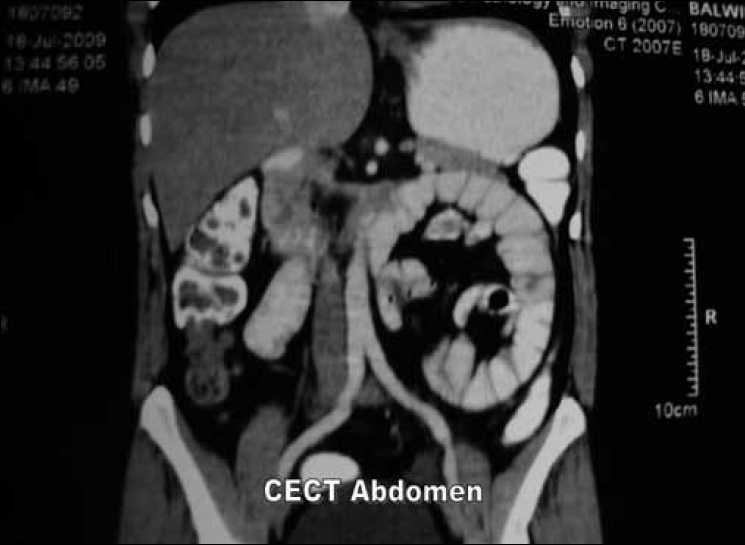
Contrast-enhanced CT scan of abdomen (CECT).

**Figure 2:**
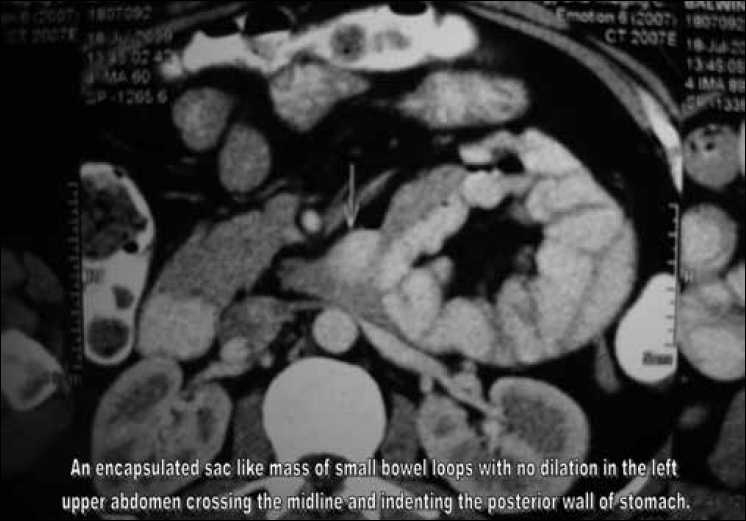
CT scan showing an encapsulated sac

Abdominal and anorectal examination, routine blood tests, x-ray abdomen, ultrasonography of abdomen, upper gastrointestinal endoscopy/ colonoscopy did not reveal any abnormality. A abdominal CT scan with oral and intravenous contrast showed an encapsulated sac–like mass of small-bowel loops with no dilatation in the left upper abdomen crossing the midline and indenting the posterior wall of the stomach [Figures 1 and 2]. A diagnosis of left paraduodenal hernia was made and the patient taken up for diagnostic laparoscopy.

**Figure 3:**
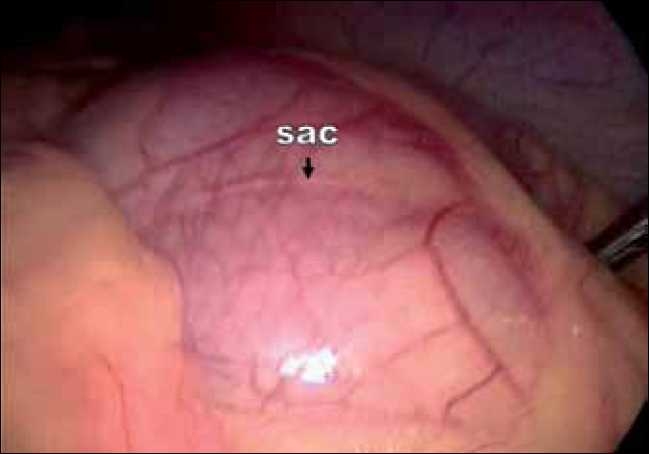
Intra-operative photograph showing the sac containing small-bowel loops.

**Figure 4:**
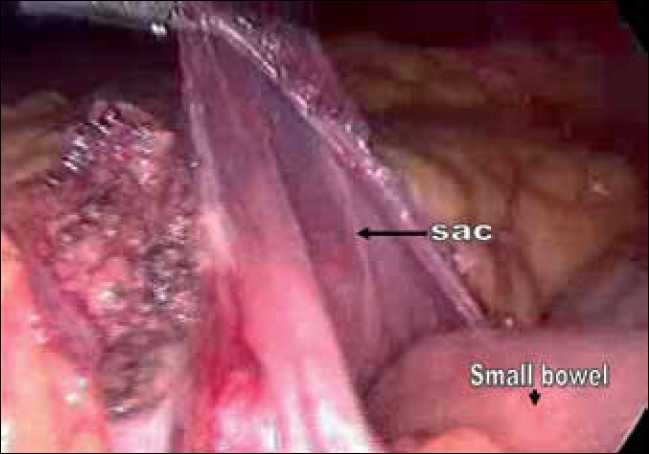
Intra-operative photograph showing the opened up sac.

**Figure 5:**
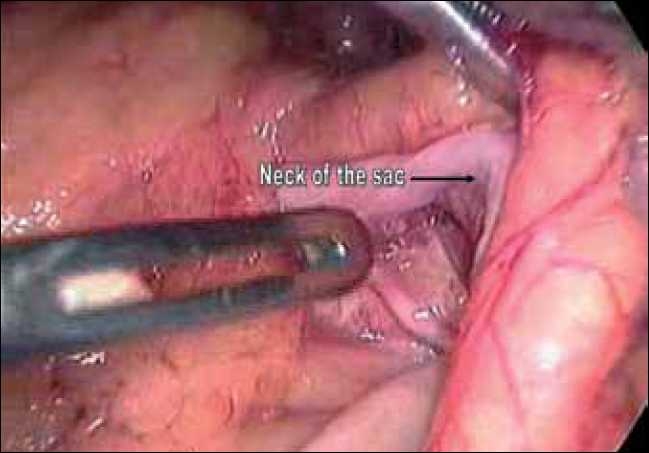
Neck of the sac.

The surgeon as well as the camera person stood on the right side of the patient. The laparoscope was inserted through a 10-mm supra-umbilical port. A 5-mm epigastric port was placed just below the xiphisternum as the left-hand working port, a 5-mm port in the right midclavicular line was used as the right-hand working port and a fourth port in the left midclavicular line below the subcostal margin was used by the assistant for traction. Intra-operatively it was observed that most of the small-bowel loops were lying within a large hernial sac [Figure 3]. The anterior wall of the hernial sac was formed by the left mesocolon. As the neck of the sac was narrow, small bowel could not be reduced. The sac was opened with an ultrasonic shears and the bowel was freed up [Figure 4]. There was no evidence of volvulus or bowel ischaemia. The neck of the sac was excised [Figure 5], and the fluid inside the sac was sampled. The patient was allowed oral fluids on postoperative day 1 and progressed to diet. He was discharged on day 3. He remains well at a follow up of six months.

## DISCUSSION

Left paraduodenal hernia is about three times more frequent than its right counterpart. It usually results from abnormal rotation of the midgut. Failure of fusion of the mesocolon with the peritoneum of the body wall leaves a potential space (the fossa of Landzert) behind the mesocolon. The left paraduodenal hernia results from invagination of the small intestine into this unsupported area through an opening bound anteriorly by the inferior mesenteric vein (IMV).[3]

Although the hernia is congenital, most patients become symptomatic between the fourth and sixth decade of life. Symptoms are non–specific, ranging from recurrent vague abdominal pain, nausea and vomiting, reversible obstruction to acute abdomen in case of incarceration and strangulation.[4] Under emergent settings, diagnosis is only possible at the time of surgery. Because of the ambiguous clinical presentation of this disease, a CT scan may be the initial tool of investigation. A characteristic finding is a cluster of small-bowel loops between the stomach and the pancreas.[5] An upper GI series with small bowel follow-through demonstrates contrast-filled loops of small bowel clustering over the left upper aspect of the abdomen.

Once diagnosed, paraduodenal hernia should be surgically repaired, because 50% of them cause intestinal obstruction.[4,6] For left-sided hernia, reduction of the bowel is fairly easy; and if there is no suggestion of bowel necrosis, laparoscopic surgery may be indicated.[2] The procedure consists of manual reduction of hernia contents followed by repair of the defect using non-absorbable sutures or incision of the hernial sac.[7] A mesh repair is reserved for large defects and recurrent hernias. If the herniated intestine is difficult to reduce because of its bulky size or adhesions within the sac, clipping and resecting the inferior mesenteric vein is helpful.[5] Although Bartlett indicated that these vessels can be divided without compromising blood supply of the colon, they should be preserved whenever possible. Intestinal resection is necessary in case of strangulation and gangrene.[8]

In right-sided hernias, the reduction of bowel from the fossa of Waldeyer formed anteriorly by the ascending mesocolon and the superior mesenteric vessels can at times be difficult even during open surgery. Care should be taken not to injure the superior mesenteric vessels during the repair.[2]

For left-sided paraduodenal hernias without bowel necrosis, laparoscopic surgery is technically easy and may be the optimum surgical method.[9] The indications are expected to increase, owing to imaging techniques allowing accurate preoperative diagnosis and as a result of advances in laparoscopic surgery.[9]

## References

[1] Huang YM, Chou AS, Wu YK, Wu CC, Lee MC, Chen HT (2005). Left Paraduodenal hernia presenting as recurrent small bowel obstruction. World J Gastroenterol.

[2] Kim JC, Kim MD, Jeong BG (2001). CT findigs of Rt. Paraduodenal Hernia Presenting as acute small bowel obstruction. J Korean Radiol soc.

[3] Brigham RA, Fallon WF, Saunders JR, Harmon JW, d’Avis JC (1984). Paraduodenal hernia: Diagnosis and surgical management. Surgery.

[4] Newsom BD, Kukora JS (1986). Congenital and acquired internal hernias; unusual causes of small bowel obstruction. Am J Surg.

[5] Olazabal A, Guasch I, Casas D (1992). Case report: CT diagnosis of nonobstructive left paraduodenal hernia. Clin Radiol.

[6] Isabel L, Birrell S, Patkin M (1995). Paraduodenal Hernia. Aust N Z J Surg.

[7] Kurachi K, Nakamura T, Hayashi T, Asai Y, Kashiwabara T, Nakajima A (2006). Left paraduodenal hernia in an adult complicated by ascending colon cancer: A case report. World J Gastroenterol.

[8] Masaki F, Akio K (2004). Laparoscopic Surgery of Left Paraduodenal Hernia. J Laparoendosc Adv Surg Tech.

[9] Bartlett MK, Wang C, Williams WH (1968). The surgical management of paraduodenal hernia. Ann Surg.

